# Noise-induced modality-specific pretext learning for pediatric chest X-ray image classification

**DOI:** 10.3389/frai.2024.1419638

**Published:** 2024-09-05

**Authors:** Sivaramakrishnan Rajaraman, Zhaohui Liang, Zhiyun Xue, Sameer Antani

**Affiliations:** Computational Health Research Branch, National Library of Medicine, National Institutes of Health, Bethesda, MD, United States

**Keywords:** chest radiography, deep learning, pediatric, modality-specific knowledge transfer, pretext learning, ensemble learning, statistical significance

## Abstract

**Introduction:**

Deep learning (DL) has significantly advanced medical image classification. However, it often relies on transfer learning (TL) from models pretrained on large, generic non-medical image datasets like ImageNet. Conversely, medical images possess unique visual characteristics that such general models may not adequately capture.

**Methods:**

This study examines the effectiveness of modality-specific pretext learning strengthened by image denoising and deblurring in enhancing the classification of pediatric chest X-ray (CXR) images into those exhibiting no findings, i.e., normal lungs, or with cardiopulmonary disease manifestations. Specifically, we use a *VGG-16-Sharp-U-Net* architecture and leverage its encoder in conjunction with a classification head to distinguish normal from abnormal pediatric CXR findings. We benchmark this performance against the traditional TL approach, *viz.*, the VGG-16 model pretrained only on ImageNet. Measures used for performance evaluation are balanced accuracy, sensitivity, specificity, F-score, Matthew’s Correlation Coefficient (MCC), Kappa statistic, and Youden’s index.

**Results:**

Our findings reveal that models developed from CXR modality-specific pretext encoders substantially outperform the ImageNet-only pretrained model, *viz.*, Baseline, and achieve significantly higher sensitivity (*p* < 0.05) with marked improvements in balanced accuracy, F-score, MCC, Kappa statistic, and Youden’s index. A novel attention-based fuzzy ensemble of the pretext-learned models further improves performance across these metrics (Balanced accuracy: 0.6376; Sensitivity: 0.4991; F-score: 0.5102; MCC: 0.2783; Kappa: 0.2782, and Youden’s index:0.2751), compared to Baseline (Balanced accuracy: 0.5654; Sensitivity: 0.1983; F-score: 0.2977; MCC: 0.1998; Kappa: 0.1599, and Youden’s index:0.1327).

**Discussion:**

The superior results of CXR modality-specific pretext learning and their ensemble underscore its potential as a viable alternative to conventional ImageNet pretraining for medical image classification. Results from this study promote further exploration of medical modality-specific TL techniques in the development of DL models for various medical imaging applications.

## 1 Introduction

In the evolving landscape of artificial intelligence (AI) driven medical diagnostics, the use of deep learning (DL) methods for chest X-ray (CXR) analysis promises enhanced clinical outcomes and operational efficiencies (Pasa et al., 2019). Chest radiography, despite its relatively lower sensitivity compared to advanced modalities like computed tomography (CT), remains a *de facto* standard in diagnostic imaging due to its cost-effectiveness, lower radiation dose, and widespread availability. Its use is particularly critical in resource-constrained environments where access to high-end imaging technologies is limited. Modern DL-driven AI leverages multi-layered neural networks to learn hierarchical feature representations directly from the data bypassing the need for manual feature extraction.

### 1.1 Related works

The application of DL models, such as convolutional neural networks (CNNs), for analyzing CXR datasets has yielded promising results in identifying, grading, and localizing cardiopulmonary manifestations such as tuberculosis, pneumonia, and COVID-19, among others, thereby enhancing the interpretative depth of automated radiographic analyses (Akhter et al., 2023; Mei et al., 2022). The advent of extensive CXR datasets, coupled with advances in DL model architecture, training strategies, and data engineering (Kumarasinghe et al., 2022; Nimalsiri et al., 2023; Kolonne et al., 2021), has catalyzed significant breakthroughs in the field of medical diagnostics. Many publicly available DL models are conventionally trained on ImageNet (Fei-Fei et al., 2010), which is an extensive collection of stock photography images. In conventional transfer learning (TL), the feature representations learned by these DL models are subsequently transferred and fine-tuned for a downstream task. *Medical modality-specific knowledge transfer*, on the other hand, refers to the strategic process of transferring knowledge gained from training a DL model on large, medical modality-specific (e.g., CXRs) datasets, encapsulating unique characteristics of those medical images, to improve performance on the related downstream task (Xue et al., 2023). Recent reviews, such as the comprehensive analysis provided in Meedeniya et al. (2022), highlight the progress in DL-based CXR analysis but also underline the need for innovations that tailor these approaches more closely to specific medical modalities. Medical imaging modalities, such as CXRs, capture various anatomical and functional information. Unlike the images in the ImageNet collection, CXRs are characterized by a high degree of similarity across different classes of pathologies and a relatively low variance within the same disease class that is separated only by subtle differences in texture, shape, and visual features. This distinction prompts a departure from using conventional TL methods toward a consideration of a medical modality-specific knowledge transfer that closely aligns with the inherent complexities of medical images (Yang et al., 2023). This approach not only addresses the limitations posed by conventional TL that learn irrelevant feature representations but also significantly reduces the risk of model overfitting, particularly when the downstream task involves using smaller and potentially imbalanced datasets that are common in medical tasks. Each of these studies contributes to the broader understanding of chest X-ray analysis but also highlights the need for more specialized approaches, particularly for pediatric populations. Our research fills this gap by implementing modality-specific learning strategies that are explicitly designed for and validated on pediatric chest X-ray datasets, addressing both the unique challenges and the clinical requirements of this demographic.

In this research, we aim to hypothesize that the CXR modality-specific pretext learning tasks such as image denoising and deblurring may help learn robust feature representations that can subsequently be transferred and fine-tuned to improve sensitivity in a downstream pediatric CXR classification task. We aim to prove that through comparison with a traditional fine-tuning approach using an ImageNet-pretrained model. To the best of our knowledge, this study is the first to attempt to perform such an analysis. Using a U-Net framework (described in Section 2.3.1), our method employs two specific pretext learning strategies. These involve the removal of Gaussian noise and Gaussian blur distortions that have been artificially applied to CXRs. We hypothesize that training the U-Net to restore clean images would force the model to learn to identify and prioritize the reconstruction of critical features over the distortions. In the process of learning the restoration, the trained models could learn to discern and encode the relevant disease feature representations in the original images. We anticipate a marked improvement in sensitivity with these CXR modality-specific pretext-learned models due to improved initialization over a naive ImageNet-only pretrained model by truncating them at the encoder and adding classification layers for a downstream pediatric CXR classification task.

### 1.2 Study contribution

The following points highlight the contributions of this study:

- We introduce an *Attention Fuzzy (A-F) ensemble* that integrates the pretext-learned models using a learnable fuzzy-based logic. By incorporating a learnable parameter that controls the fuzziness of the model combination, we aim to strike a balance, dynamically adjusting the influence each model exerts based on its confidence.
- Our research demonstrates the effectiveness of leveraging the specialized knowledge gained from CXR denoising and deblurring tasks to enhance performance in a downstream CXR classification task over conventional TL approaches.
- We adopt a U-Net model that is traditionally utilized for biomedical image segmentation for the CXR denoising and deblurring pretext learning tasks, demonstrating its versatility.

As a result of these contributions, we believe that our study lays the groundwork for broader applications of medical modality-specific knowledge transfer for other DL-based medical image analyses.

## 2 Materials and methods

### 2.1 Datasets and preprocessing

The datasets used in our study include the following:

- Pediatric Pneumonia CXR (Kermany et al., 2018): A collection of de-identified images from the Guangzhou Medical Center in China comprising 4,273 CXRs of children aged 1–5 years that are diagnosed with either bacterial or viral pneumonia and 1,583 CXRs of normal (no-findings) lungs. We use this dataset for image denoising and deblurring tasks.
- VinDr-PCXR (Pham et al., 2023): This dataset includes 9,125 de-identified CXR scans collected from major Vietnamese hospitals between 2020 and 2021. The collection features CXR images of 5,354 male and 3,709 female pediatric patients with 62 pediatric images of undisclosed gender. Out of 8,755 pediatric CXRs, 5,876 show normal lungs while 2,879 manifest various cardiopulmonary abnormalities. We use this dataset for the classification task.

Both datasets are split at the patient level into three groups: 70% for training, 10% for validation, and 20% for hold-out testing, respectively, to prevent data leakage and learning bias. Table 1 provides the details of this data partition.

**Table 1:** Dataset partitioning.

Dataset	Task	Total	Training	Validation	Test
Pediatric Pneumonia CXR	Denoising/deblurring	5,856	4,100	586	1,170
VinDr-PCXR	Classification	8,755	6,129	876	1,750

This table shows the total number of images in the two datasets used in this study with respective fractions used for training, validation, and hold-out testing.

We apply a U-Net model with an Inception-V3 encoder backbone, which we previously used in Rajaraman et al. (2023) to segment the lung regions and crop them to a bounding box encapsulating the lung pixels. This approach prevents the model from learning features that are irrelevant to the lung regions in CXR images. The cropped images are resized to 224 × 224 pixels to address computational demands and the pixel values are normalized to the range [0, 1].

### 2.2 Adding Gaussian noise and blur

The quality of the CXR image plays a pivotal role in their automated diagnostic analysis and accurate disease identification. Achieving optimal image quality is often challenged by various factors, including technical limitations, patient-specific conditions, and the inherent constraints of imaging modalities. One such critical challenge is radiation underexposure, a frequent occurrence in clinical settings, which introduces quantum mottle or noise (Huda and Abrahams, 2015; Tischenko et al., 2005) and significantly affects their diagnostic utility. We adopt a Gaussian noise addition approach to simulate this noise and mimic the effects typically observed in CXRs exhibiting radiation underexposure.

- Each CXR image is processed to normalize its intensity levels to a range between 0 and 1. This standardization is crucial for ensuring consistency in the subsequent noise addition process across all images.
- We generate Gaussian noise characterized by a zero mean and a unit standard deviation. This noise is scaled by a set of predefined variance factors [0.02, 0.04, 0.06, 0.08, 0.1], representing different levels of radiation underexposure. The variance factors are chosen to span a realistic spectrum of underexposure conditions, from mild to severe.
- For each image in the Pediatric Pneumonia CXR dataset, a variance factor is randomly selected from this predefined list. This ensures that each image is subjected to a unique level of noise, thereby introducing variability into the training process.
- The synthesized noise is added to the normalized CXR images with the noise intensity tailored per the chosen variance factor.
- Finally, the images added with Gaussian noise are rescaled to their original intensity range of 0–255 and saved. This step ensures the retention of the original image format while incorporating the simulated effects of noise.

The clarity of a CXR is also influenced by the capabilities of the imaging detector. A phenomenon known as detector crosstalk occurs when the energy from one pixel inadvertently spreads to those nearby, leading to blurring (Huda and Abrahams, 2015). Movements or vibrations, whether from the subject being imaged or the imaging apparatus itself, also add to the blur, further complicating the task of obtaining clear images. To simulate these, we adopt a Gaussian blurring approach to mimic such effects observed in CXR images. The methodology utilized for applying Gaussian blur is discussed below:

- We apply Gaussian blur, characterized by its kernel size, that determines the extent of blurring. The kernel size is selected randomly from a predefined set of sizes [3 × 3, 5 × 5, 7 × 7, and 9 × 9].
- The standard deviation (sigma) of the Gaussian filter is automatically calculated using the function in the OpenCV library such that the blurring effect is optimally adjusted according to the kernel size.
- The processed images with Gaussian blur applied are saved for subsequent analysis.

It is crucial to acknowledge the stochastic nature of noise and blur phenomena in clinical imaging settings. In real-world scenarios, blur and noise do not follow a uniform or deterministic pattern; they are subject to a wide range of variabilities due to patient movement, variations in imaging protocols and radiographer skill, and intrinsic properties of the detector systems. To simulate this randomness and its impact on imaging quality, we incorporate a stochastic component in our methodology by introducing random variations in the noise and blur applied to each image. This random selection from our predefined set of variance factors for the noise and the kernel sizes for the blur aims to more closely mimic the unpredictable and varied nature of noise and blur encountered in clinical practice. It is important to note that the literature does not explicitly define the direct correlation between specific variance factors and the noise and the Gaussian filter sizes and the blur present in CXRs. Our approach is a methodological choice that aims to create a dataset that challenges the robustness of the algorithm against various degrees of image degradation, thereby introducing variability, rather than precise modeling of the physical phenomena of noise and blur.

### 2.3 Model architecture

#### 2.3.1 VGG-16-Sharp-U-Net

We use the *VGG-16-Sharp-U-Net* architecture for image denoising and deblurring tasks. The encoder, or the contracting path, adopts the VGG-16 (Simonyan and Zisserman, 2015) architecture and is initialized with random weights. The VGG-16 model, renowned for its simplicity and effectiveness, has been widely adopted in the field of medical image classification, particularly with CXRs (Bougias et al., 2021; Nishio et al., 2020). The 1st and 2nd encoder block consists of two 3 × 3 convolutional layers with rectified linear unit (ReLU) activations, followed by a 2 × 2 max-pooling layer. The 3rd, 4th, and 5th encoder blocks consist of three 3 × 3 convolutional layers with ReLU activations, followed by a 2 × 2 max-pooling layer, except the 5th block, which does not include a pooling layer. We use 64, 128, 256, 512, and 512 filters, respectively, for the convolutional layers in the encoder blocks. The symmetrical decoder or the expanding path may be regarded as an operator that performs the reverse of the contracting path. The 1st and 2nd block in the decoder comprises a 2 × 2 up-convolution to up-sample the features, followed by three 3 × 3 convolutions with ReLU activations. The 3rd and 4th blocks comprise a 2 × 2 up-convolution, followed by two 3 × 3 convolutions with ReLU activations. The final convolutional layer has one 3 × 3 convolution and a sigmoidal activation to predict the denoised/deblurred image. The encoder abstracts and compresses the input data. This leads to the loss of fine-grained details crucial for precise localization. The decoder projects the lower-resolution encodings back to the original image space. However, solely relying on the decoder’s up-sampled outputs can result in a loss of detail due to the prior compression steps. Skip connections mitigate information loss by concatenating the high-resolution features from the encoder to the corresponding up-sampled features in the decoder. This fusion of context and localization cues enables the U-Net to reconstruct images with both high-level semantic clarity and detailed spatial accuracy. The authors of Zunair and Ben (2021) observe that a feature mismatch occurs when the low-level, fine-grained encoder features are fused with the high-level, semantic, and course-grained decoder features. This fusion may result in blurred feature maps throughout the learning process and may adversely affect image reconstruction. Hence, Zunair and Ben (2021) proposed an approach to reduce this feature mismatch where the encoder features undergo a depth-wise convolution (i.e., spatial convolution operation performed independently over each channel of the encoder features) with a sharpening spatial kernel. This sharpening operation enhances edges and details, thereby sharpening the details in the encoder feature maps and hence balancing the semantic gap introduced by the high-level process in the decoder network. By making the encoder and decoder features more semantically compatible, the sharpening operation facilitates a smoother gradient flow across the network during backpropagation (Zunair and Ben, 2021). The sharpening operation does not introduce any learnable parameters. Such an approach might be especially beneficial in learning denoising and deblurring tasks, as these tasks necessitate the preservation and emphasis of fine image details that are often compromised by noise and blur artifacts. The sharpened feature maps are thus more aligned in semantic richness with the decoder’s outputs, facilitating a more coherent and detailed reconstruction of the denoised or deblurred image.

**Figure 1:**
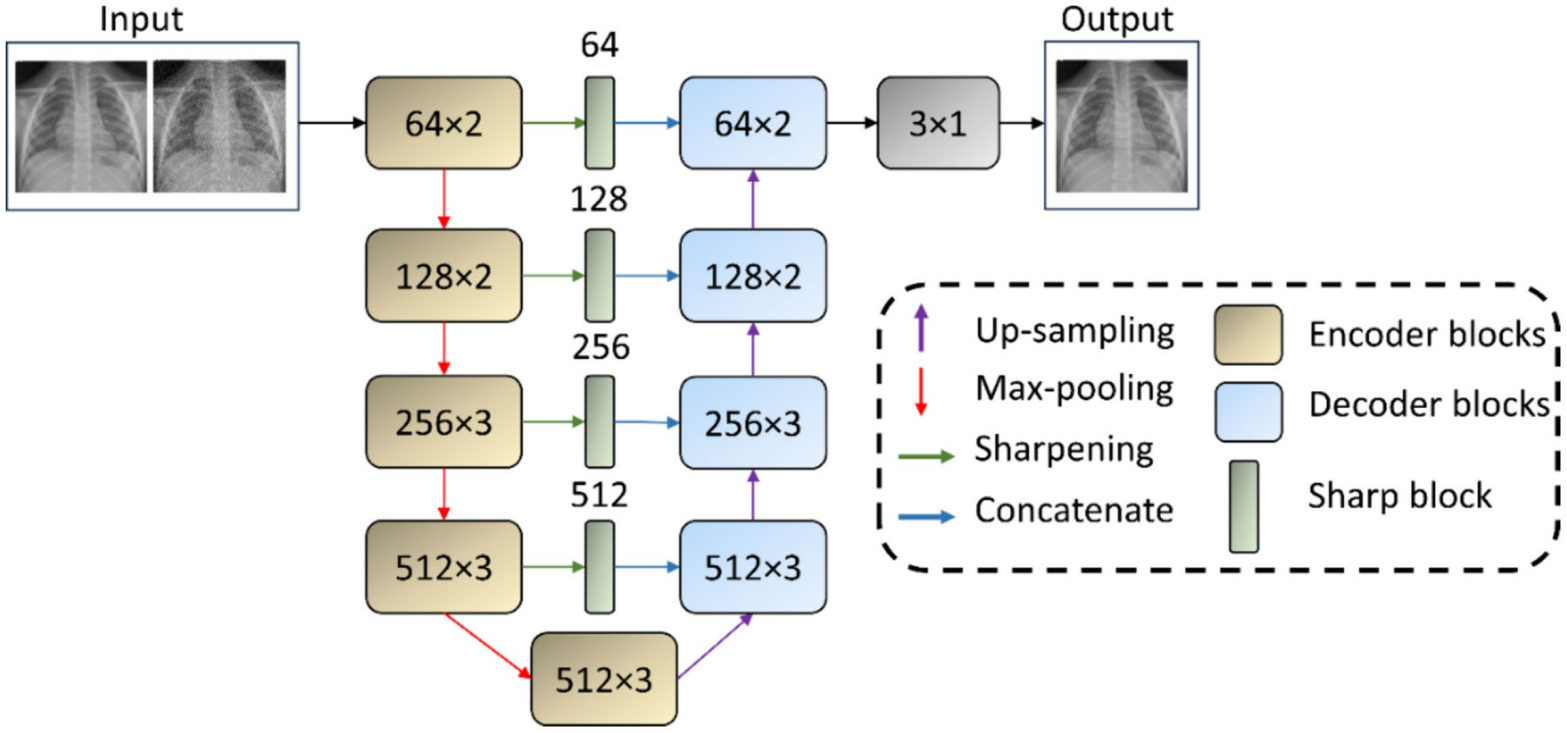
*VGG-16-Sharp-U-Net* architecture. The input is a Gaussian noise-added image with its original counterpart as the label. The model predicts a denoised image. A similar architecture is used with a Gaussian blur-added image as the input with its original counterpart as the label. The model then predicts a deblurred image.

Using the interactive tool at Setosa.io^1^ to study the effects of various image kernels, we elected to incorporate a 3 × 3 sharpening kernel in the skip connections between the encoder and the decoder. A sharpening kernel, defined as [0 –1 0; −1 5 –1; 0 –1 0], was specifically selected for its capacity to accentuate the differences in adjacent pixel values, a property that is essential in medical imaging for enhancing edge definition and contrast. The central weight of 5 in the sharpening kernel increases the intensity of the center pixel relative to its neighbors. This is particularly effective for maintaining and highlighting critical structures within the images, such as edges, textures, and fine details. The negative weights surrounding the center serve to subtract the average surrounding pixel value, further accentuating the differences between adjacent pixel intensities. This operation effectively highlights edges and textures by increasing their visibility against the surrounding areas, thereby enhancing the perceptual sharpness of the image. Depth-wise convolutions process each channel of the input independently, preserving the distinct characteristics of each feature map while applying the sharpening effect. Figure 1 shows the architecture of the *VGG-16-Sharp-U-Net* used in our study.

The proposed model is trained with image pairs. For the denoising task, the input is a Gaussian noise-added image with its original, clean counterpart as the label. For the deblurring task, the input is a Gaussian-blurred image with its clean counterpart as the label. The model is configured to output a denoised/deblurred image. We utilize the Adam optimizer for training optimization, starting with an initial learning rate of 1 × 10^−3^. We minimize the Structural Similarity Index Measure (SSIM) loss, which is chosen for its efficacy in capturing the perceptual difference between the predicted and ground-truth (GT) images. We monitor Mean Squared Error (MSE) and Mean Absolute Error (MAE) as secondary metrics, along with the Peak Signal-to-Noise Ratio (PSNR) and SSIM for performance evaluation. We implement a robust training regimen complemented by several key callback functions aimed at optimizing the training process and ensuring model generalization. We dynamically adjust the learning rate during training while monitoring the validation loss as a key indicator of model performance. If the validation loss ceases to decrease for 10 epochs, a scenario indicative of a plateau in the learning process, the learning rate is reduced by a factor of 0.5. This approach allows the model to make finer adjustments to the weights, potentially navigating out of local minima or plateaus in the loss landscape. The learning rate reduction continues until it reaches a lower bound of 1 × 10^−6^, ensuring that the learning rate does not decrease to a level that would be counterproductive for the training process. Concurrently, we save only the model weights that achieve the lowest validation loss, thereby ensuring that the model version with the highest generalization capability is retained for subsequent evaluation.

#### 2.3.2 Classification models

The encoder of the trained denoising and deblurring models is truncated at the deepest convolutional layer. This is followed by the addition of classification-specific layers, which include a global average pooling (GAP) layer and a dense output layer with two nodes and a Softmax activation function. The adapted models, hereinafter called *denoising pretext (Den-P)* and *deblurring pretext (Deb-P)*, are fine-tuned to fulfill the objective of classifying pediatric CXRs in the VinDr-PCXR dataset as those manifesting cardiopulmonary diseases and those without such signs, categorizing them as normal. To establish a benchmark for performance evaluation, a *baseline (B)* model is constructed by truncating an ImageNet pre-trained VGG-16 model at its deepest convolutional layer, followed by the addition of a GAP layer and a dense output layer with two nodes and a Softmax activation function. This model is then fine-tuned to parallel the classification capability of the *Den-P* and *Deb-P* models.

#### 2.3.3 Ensemble learning

CNNs learn through error backpropagation and stochastic optimization to minimize loss for accurate image categorization. However, training data inconsistencies may lead to potential overfitting and increased prediction variance, which may adversely impact their performance. To mitigate these challenges, we investigate ensemble learning (Asgharnezhad et al., 2022; Rao et al., 2024), a strategy that amalgamates multiple, distinct CNNs to harness their collective intelligence, thereby diminishing prediction variance. In medical imaging, particularly in classifying CXRs, ensemble models have shown considerable promise, employing techniques like majority voting, and simple and weighted averaging (Chowdhury et al., 2021; Rajaraman et al., 2020; Abedalla et al., 2021). Our methodology encompasses constructing ensemble predictions from *Den-P* and *Deb-P* models, utilizing both simple averaging (*SA*) and Sequential Least-Squares Programming (SLSQP)-based (Marques et al., 2021) weighted averaging (*SLSQP-WA*). A simple averaging ensemble involves averaging the output probabilities of the individual models. Mathematically, if $p_{i}\left(x\right)$ is the prediction of the ‘ith’ model, the final prediction p(x) is computed as given by Equation 1. Here, N denotes the number of models. Simple averaging is straightforward and often effective in reducing variance without the need for complex weighting schemes.

(1) $$p\left(x\right)=\frac{1}{N}\overset{N}{\underset{i=1}{\sum }}p_{i}\left(x\right).$$

Sequential Least-Squares Programming (SLSQP)-based Weighted Averaging is an advanced technique that optimizes the weights assigned to each model’s predictions to minimize logarithmic loss. The weights are found by solving the following optimization problem, as shown in Equation 2. SLSQP provides a numerically efficient method to perform this optimization, adapting weights based on the reliability and performance of individual models.

(2) $$\min_{w}\overset{N}{\underset{i=1}{\sum }}w_{i}\log\left(p_{i}\left(x\right)\right)\mathrm{subject to}\overset{N}{\underset{i=1}{\sum }}w_{i}=1,w_{i}\geq 0.$$

We propose a novel model ensemble, hereafter called the *Attention Fuzzy (A-F)* ensemble, that integrates the *Den-P* and *Deb-P* models using a learnable fuzzy-based logic (Ieracitano et al., 2022). The mathematical model for the A-F ensemble is expressed as shown in Equation 3. Here, p(x) denotes the final prediction where $\alpha_{i}$ are the attention weights applied to the logits, $f_{i}\left(x\right)$ , from each model, combined and passed through a softmax function for normalization.

(3) $$p\left(x\right)=\mathrm{softmax}\left(\overset{N}{\underset{i=1}{\sum }}\alpha_{i}f_{i}\left(x\right)\right).$$

By incorporating a learnable parameter that controls the *fuzziness* of the model combination, we aim to strike a balance, dynamically adjusting the influence each model exerts based on its confidence. We load the *Den-P* and *Deb-P* models and freeze their trainable layers. The features from their deepest convolutional layer are extracted and fed into their respective GAP layers. The feature maps from the GAP layers are then concatenated. An attention mechanism, implemented via a trainable dense layer, assigns weights to the concatenated feature. Such an approach guides the ensemble to focus on relevant features from each model’s output. We introduce a custom layer, which multiplies the logits by a trainable *fuzziness* parameter before applying a Softmax activation. This layer introduces a controllable degree of uncertainty to the final predictions. During training, the proposed *A-F* ensemble adjusts its parameters, including the learnable *fuzziness*, according to the backpropagated errors. This learning process ensures that the *A-F* ensemble not only learns the best representations from the constituent models but also the optimal degree of *fuzziness* for the Softmax operation.

Optimization of the *baseline*, *Den-P*, *Deb-P*, and *A-F* ensemble models is carried out using the Stochastic Gradient Descent (SGD) algorithm, with an initial learning rate set at 1 × 10^−3^. The learning rate is dynamically adjusted downwards in response to periods of validation loss plateau, enhancing the training process by avoiding stagnation and promoting loss minimization. Throughout the training process, internal monitoring is implemented via callbacks to check models’ performance, while tracking improvements in validation loss. Model checkpoints are saved at instances of validation loss improvement, ensuring that the model checkpoints with the minimal validation loss are selected for final evaluations on the hold-out test set. Our experiments are conducted using Tensorflow Keras v.2.10 on an Ubuntu system with a Xeon E5-2640v3 processor, 64GB Random Access Memory (RAM), NVIDIA® 2080Ti graphical processing unit (GPU), and CUDA dependencies for GPU acceleration.

### 2.4 Activation visualization using score-weighted class activation mapping (score-CAM)

We use Score-weighted Class Activation Mapping (Score-CAM) (Wang et al., 2020) to visualize the learned behavior of the *baseline*, *Den-P*, and *Deb-P* models. Unlike conventional Class Activation Mapping (CAM) and Gradient-weighted Class Activation Mapping (Grad-CAM) methods, Score-CAM eliminates the dependence on gradients by obtaining the weight of each activation map through its forward passing score on the target class. The final result is obtained by a linear combination of weights and activation maps. Score-CAM is reported to achieve improved visual performance and fairness than conventional CAM and Grad-CAM methods for interpreting decision-making processes. We use Score-CAM to generate the activation maps from the deepest convolutional layer of the *baseline*, *Den-P*, and *Deb-P* models. These activation maps are resized to the input shape and normalized. The resulting activation maps are translated into bounding boxes by identifying their extreme points. These boxes are then overlaid on the test CXRs to directly compare with GT annotations released by Pham et al. (2023), thereby facilitating a qualitative assessment of the models’ performance.

### 2.5 Evaluation metrics

#### 2.5.1 Denoising/deblurring

We use the following metrics to evaluate the performance of the *VGG-16-Sharp-U-Net* model trained for denoising and deblurring tasks: (a) Peak Signal-to-Noise Ratio (PSNR); (b) Structural Similarity Index (SSIM), and (c) Haar Wavelet-based Perceptual Similarity Index (HaarPSI). PSNR is a widely used metric to measure the quality of reconstruction as it provides a clear, quantitative measure of reconstruction error. It compares the similarity between the GT and predicted image based on their MSE, given by Equation 4.

(4) $$PSNR=10\cdot \log_{10}\left(\;\frac{MAX_{I}^{2}}{MSE}\right).$$

Here, $MAX_{I}^{2}$ is the maximum possible pixel value of the image (e.g., 255 for 8-bit images), and *MSE* is the mean squared error between the GT and the predicted image. The *MSE* value is given by Equation 5.

(5) $$MSE=\frac{1}{m\times n}\left(\overset{m-1}{\underset{i=0}{\sum }}\overset{n-1}{\underset{j=0}{\sum }}{\left[I\left(i,j\right)-K\left(i,j\right)\right]}^{2}\right).$$

Here, I is the original image, K is the predicted denoised/deblurred image, and m,n are the dimensions of the images. PSNR is typically measured in decibels (dB). The range of PSNR is from 0 to infinity, theoretically. In practice, PSNR values commonly fall between 20 dB and 50 dB for image processing applications. Higher PSNR values indicate better quality of the predicted image.

SSIM measures the perceived quality of an image by comparing local patterns of pixel intensities that have been normalized for luminance and contrast. SSIM is particularly useful to compare model performance in tasks including denoising and deblurring as it considers texture, contrast, and structure in its evaluation, thereby offering a more comprehensive assessment of image quality and similarity to human perception than PSNR. The SSIM measured for a pair of images (x,y) is given by Equation 6.

(6) $$SSIM\left(x,y\right)=\frac{\left(2\mu_{x}\mu_{y}+C_{1}\right)\left(2\sigma_{xy}+C_{2}\right)}{\left(\mu_{x}^{2}+\mu_{y}^{2}+C_{1}\right)\left(\sigma_{x}^{2}+\sigma_{y}^{2}+C_{2}\right)}.$$

Here, $\mu_{x},\mu_{y}$ are the average intensities, $\sigma_{x}^{2},\sigma_{y}^{2}$ are the variances, $\sigma_{xy}$ is the covariance of images xandy , $C_{1}$ and $C_{2}$ are constants used to maintain stability. SSIM values range from −1 to 1. A value of 1 indicates perfect similarity between the GT and the predicted image, implying no distortion. Values closer to −1 indicate a lack of similarity, signifying significant distortion or differences between the GT and the predicted image.

HaarPSI (Reisenhofer et al., 2018) is a performance metric designed to assess the perceptual similarity between two images. The computational steps involved in HaarPSI are listed as follows: (i) Local similarity evaluation: This step involves the use of high-frequency Haar wavelet coefficients to compare local areas between the GT and predicted images, focusing on capturing details like edges and textures that are crucial for human perception; (ii) Importance Weighting: The low-frequency Haar wavelet coefficients are used to determine the importance of each area within the image, assuming that not all discrepancies have an equal impact on the perceived image quality. This approach ensures reflecting the human visual system’s varying sensitivity to different image areas, providing a more nuanced assessment of image quality improvements or degradations. While the authors of Reisenhofer et al. (2018) provide an exhaustive mathematical formulation, a simplified representation can be conceptualized as in Equation 7.

(7) $$HaarPSI=\frac{\sum \left(Similarityscores\times Importanceweights\right)}{\sum \left(Importanceweights\right)}.$$

The final HaarPSI score ranges from 0 to 1, where 1 indicates perfect perceptual similarity and 0 indicates no perceptual similarity between the GT and predicted images.

#### 2.5.2 Classification

The following metrics are used to evaluate the classification performance: (i) Balanced accuracy; (ii) Sensitivity; (iii) Specificity; (iv) F-score (F); (v) Matthews correlation coefficient (MCC); (vi) Kappa statistic, and (vii) Youden’s index. While these metrics are widely discussed and utilized in literature (Erickson and Kitamura, 2021), balanced accuracy receives comparatively less attention. While conventional accuracy can provide a quick overview of model performance, it may not always offer a fair assessment, especially in imbalanced datasets. In such cases, a model might heavily favor the majority class, leading to high overall accuracy while performing poorly on the minority class. Hence, balanced accuracy is an important measure to use in scenarios involving imbalanced datasets. Balanced accuracy is calculated as the average of sensitivity and specificity achieved in each class, computed at a classification threshold of 0.5. The sensitivity for a class is defined as the ratio of correctly predicted positive observations to all observations in that actual class. Balanced accuracy is given by Equation 8.

(8) $$Balancedaccuracy=\frac{1}{2}\left(\frac{TP}{TP+FN}+\frac{TN}{TN+FP}\right).$$

Here, True Positives ( TP ) are correctly predicted positive values, True Negatives ( TN ) are the correctly predicted negative values, False Positives ( FP ) are negative values that are incorrectly predicted as positive, and False Negatives ( FN ) are positive values that are incorrectly predicted as negative.

### 2.6 Statistical analysis

We perform statistical analysis to assess the differences in the sensitivity between various computational models under study. The rationale for this comparative analysis is to statistically determine whether the improvements in sensitivity are significant or occur by chance, thereby informing the selection of the most effective model for the current classification task. For each model, the sensitivity and the corresponding 95% confidence intervals (CI) are established. The standard error (*SE*) of sensitivity is calculated as shown in Equation 9.

(9) $$SE=\frac{\left(CI_{upper}-CI_{lower}\right)}{2*1.96}.$$

Here, $CI_{upper}\;\mathrm{and}\;CI_{lower}$ denote the upper and lower bounds of the *CI*. Next, we compute the differences in sensitivity (*ΔSensitivity*), as in Equation 10, between pairs of classification models and their associated combined standard error (*ΔSE*), as in Equation 11, which incorporates the variability of both models being compared.

(10) ΔSensitivity=Sensitivity2−Sensitivity1.

(11) $$\Delta SE=sqrt\left(SE1^{2}+SE2^{2}\right).$$

Here, *Sensitivity1* and *Sensitivity2* are the sensitivity metrics*, and SE1* and *SE2* are the SE metrics of the compared models. To determine the statistical significance of the differences in sensitivity between each pair of models, we computed the Z-score (*Z*) using Equation 12.

(12) Z=ΔSensitivity/ΔSE.

The *Z*-score represents how many standard deviations the observed difference in sensitivity is away from the null hypothesis (i.e., no sensitivity difference between the compared models). We evaluate the corresponding two-tailed *p*-value (*p*) for each *Z*-score based on the standard normal distribution. The statistical significance level is set to 0.05. A low *p*-value (<0.05) indicates a statistically significant difference in sensitivity. The analysis is conducted using Python with NumPy for numerical operations and SciPy for statistical functions. A series of pairwise comparisons were made between six models, i.e., Baseline (*B*), *Den-P*, *Deb-P*, *SA*, *SLSQP-WA*, and the *A-F* ensemble. The sensitivity difference, SE, and *p*-values for each comparison are visualized in a graph.

## 3 Results and discussion

### 3.1 Denoising/deblurring performance analysis

The *VGG-16 Sharp-U-Net* model’s performance in the tasks of denoising and deblurring the pediatric CXR images is quantitatively assessed using PSNR, SSIM, and HaarPSI metrics. Table 2 shows the performance achieved by the denoising and deblurring models.

**Table 2:** Performance of denoising and deblurring models.

Task	PSNR	SSIM	HaarPSI
Denoising	29.7535	0.6578	0.7909
Deblurring	**39.7211**	**0.9700**	**0.9502**

Bold numerical values denote superior performance in respective columns.

**Figure 2:**
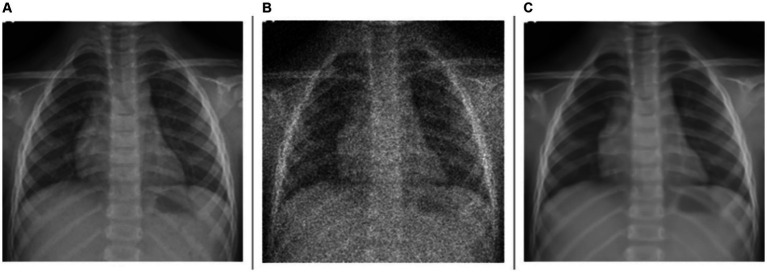
Image denoising. **(A)** Original CXR, **(B)** Gaussian noise-added CXR (variance factor = 0.08), and **(C)** predicted denoised image.

As observed from Table 2, for the denoising task, the model achieves a PSNR of 29.7535 dB, indicating a moderate level of noise reduction. The model scores an SSIM of 0.6578, reflecting an acceptable level of visual similarity to the original images post-denoising. The HaarPSI is recorded at 0.7909, illustrating a good perceptual similarity between the denoised images and their original counterparts. This is evident from Figure 2 which shows a sample instance of a pediatric test CXR with its Gaussian noise-added counterpart and the predicted denoised image.

**Figure 3:**
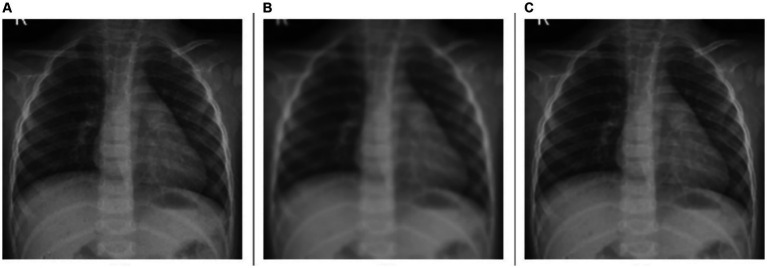
Image deblurring. **(A)** Original CXR, **(B)** Gaussian blur-added CXR (kernel size = 7), and **(C)** predicted deblurred image.

For the deblurring task, the model achieves a markedly higher value of PSNR at 39.7211 dB, indicating good performance in reducing blur and restoring image clarity. The SSIM value is 0.9700, demonstrating that the deblurred images closely match the original ones in structure, contrast, and luminance. The model achieves a HaarPSI score of 0.9502, denoting a markedly improved perceptual similarity to the original images, post-deblurring. We could visualize this prediction quality through Figure 3, showing a sample instance of a pediatric test CXR with its Gaussian blur-added counterpart and the predicted deblurred image.

A comparative analysis of the denoising and deblurring models demonstrates that the proposed *VGG-16 Sharp-U-Net* model exhibits superior performance in the deblurring task over denoising, as evidenced by higher values across all metrics. This distinction could be attributed to the inherent complexity of each task. Deblurring, focused on correcting uniform distortions, might be a more straightforward objective compared to denoising, which involves addressing random noise patterns that can vary significantly across images.

### 3.2 Classification performance analysis

**Figure 4:**
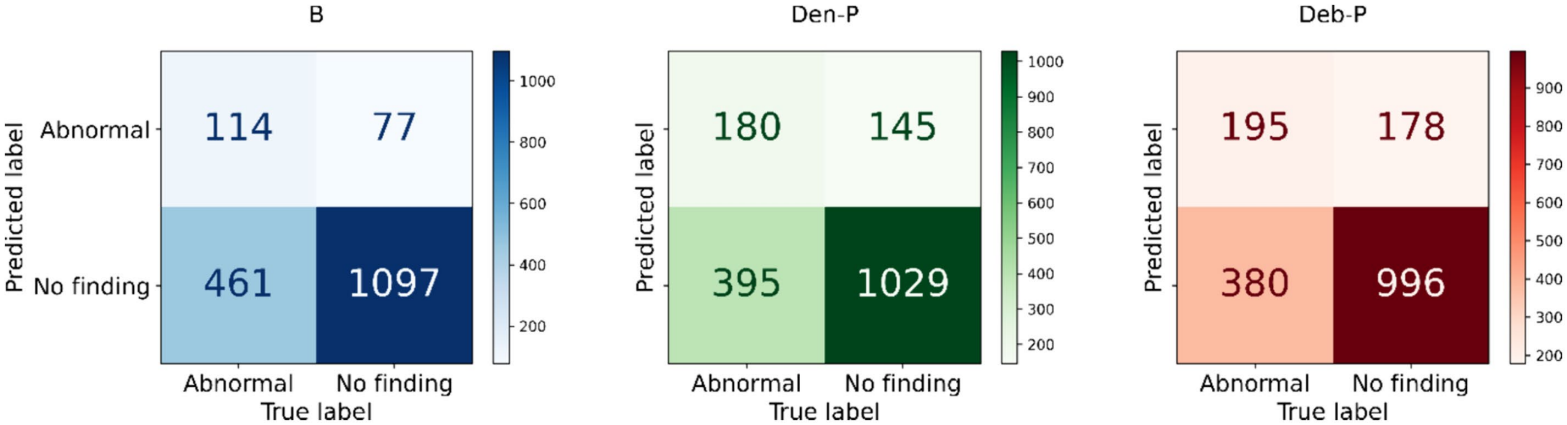
Test confusion matrices obtained with the *baseline*, denoising pretext (*Den-P*), and deblurring pretext (*Deb-P*) models.

**Figure 5:**
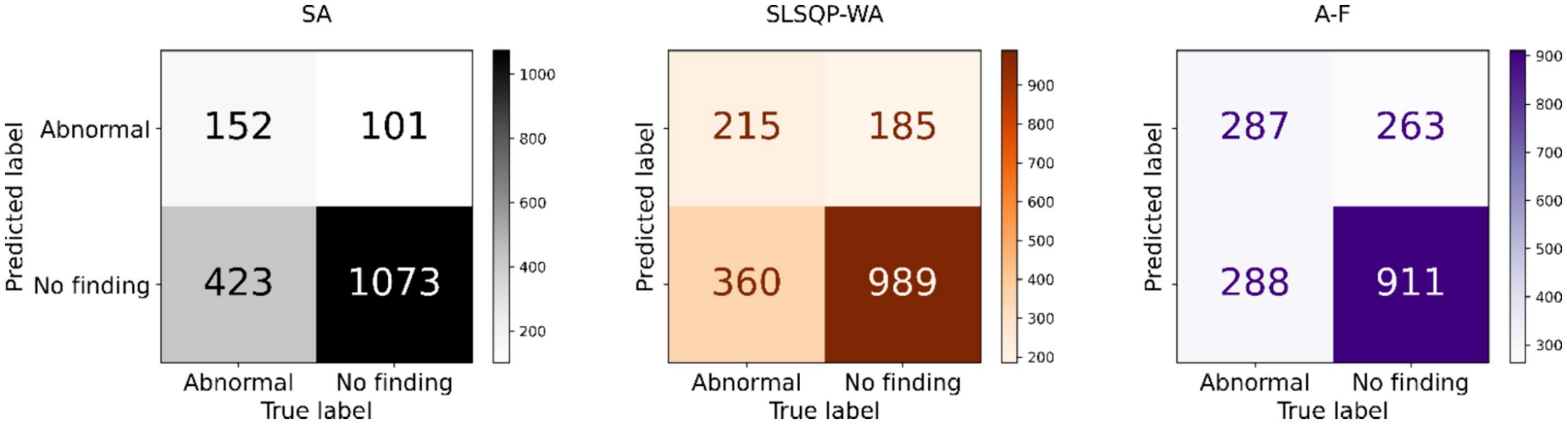
Test confusion matrices obtained with the ensembles.

**Figure 6:**
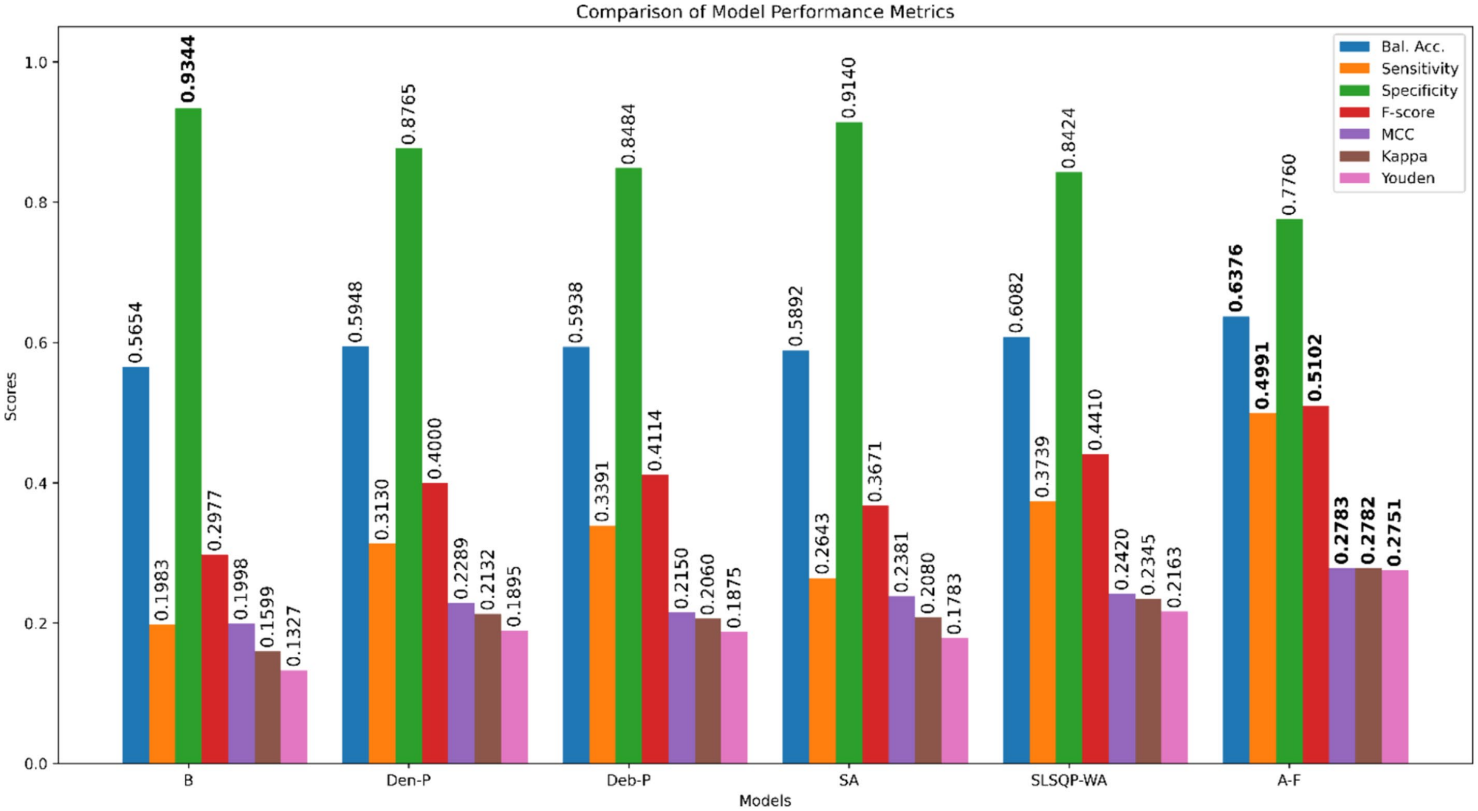
Bar plot comparing the performance of all models across the classification metrics. Bold numerical values denote superior performance for the respective performance metric.

The performance of the classification models, *viz.*, *baseline*, *Den-P*, *Deb-P*, and ensembles are shown in Table 3 and the confusion matrices are shown in Figures 4, 5. The bar plot shown in Figure 6 presents a comparative analysis of various models employed in the classification task. The *Baseline (B)* model sets the benchmark with a balanced accuracy (Bal. Acc.) of 0.5654, yet it lags in sensitivity, identifying TPs at a rate of only 0.1983. While its specificity stands high at 0.9344, suggesting a strong ability to recognize TNs, the model’s F-score, MCC, Kappa, and Youden’s index are modest, indicating room for improvement in balanced classification performance. The *Den-P* model exhibits an improvement in sensitivity to 0.3130, indicating a better detection rate of actual disease manifestations but at the expense of specificity, which drops to 0.8765. This shift suggests a trade-off between detecting more TPs and incorrectly classifying some healthy cases as diseased. Conversely, the *Deb-P* model shows a marked enhancement in sensitivity (0.3391) over the *baseline*, aligning with a moderate specificity of 0.8484. This reflects a nuanced improvement in identifying pathological features within the CXRs, although it still faces challenges in accurately segregating all healthy cases. The *Den-P* and *Deb-P* models exhibit enhanced balanced accuracy, sensitivity, F-score, MCC, Kappa, and Youden’s index compared to the *baseline* model that relied on ImageNet-pretrained weights, highlighting the benefit of CXR modality-specific pretext learning and fine-tuning in the realm of pediatric CXR classification.

**Table 3:** Performance comparison of constituent models and their ensembles.

Model	Bal. Acc.	Sensitivity	Specificity	F-score	MCC	Kappa	Youden
*B*	0.5654	0.1983	**0.9344**	0.2977	0.1998	0.1599	0.1327
*Den-P*	0.5948	0.3130	0.8765	0.4000	0.2289	0.2132	0.1895
*Deb-P*	0.5938	0.3391	0.8484	0.4114	0.2150	0.2060	0.1875
*SA*	0.5892	0.2643	0.9140	0.3671	0.2381	0.2080	0.1783
*SLSQP-WA*	0.6082	0.3739	0.8424	0.4410	0.2420	0.2345	0.2163
*A-F*	**0.6376**	**0.4991**	0.7760	**0.5102**	**0.2783**	**0.2782**	**0.2751**

The acronyms SA, SLSQP-WA, and A-F denote simple averaging, SLSQP-based weighted averaging, and Attention Fuzzy ensembles, respectively. Bold numerical values denote superior performance in respective columns.

Simple averaging of the predictions of the *Den-P* and *Deb-P* further improves specificity and MCC. The *SLSQP-WA* ensemble of the predictions of the *Den-P* and *Deb-P* models improves balanced accuracy, sensitivity, F-score, MCC, Kappa, and Youden’s index compared to the constituent models. The standout performer is the *A-F* ensemble, which demonstrates the highest balanced accuracy (0.6376) and sensitivity (0.4991), both metrics markedly superior to the individual constituent models, thereby underscoring its robustness in classification. This indicates a synergistic effect, where the *A-F* ensemble effectively combines the strengths of the individual models to achieve higher accuracy in classifying the pediatric CXRs. Moreover, the *A-F* ensemble demonstrates the highest F-score (0.5102), MCC (0.2783), Kappa (0.2782), and Youden’s index (0.2751) suggesting it is the most proficient ensemble in delivering superior performance across these metrics.

### 3.3 Performance significance analysis

**Figure 7:**
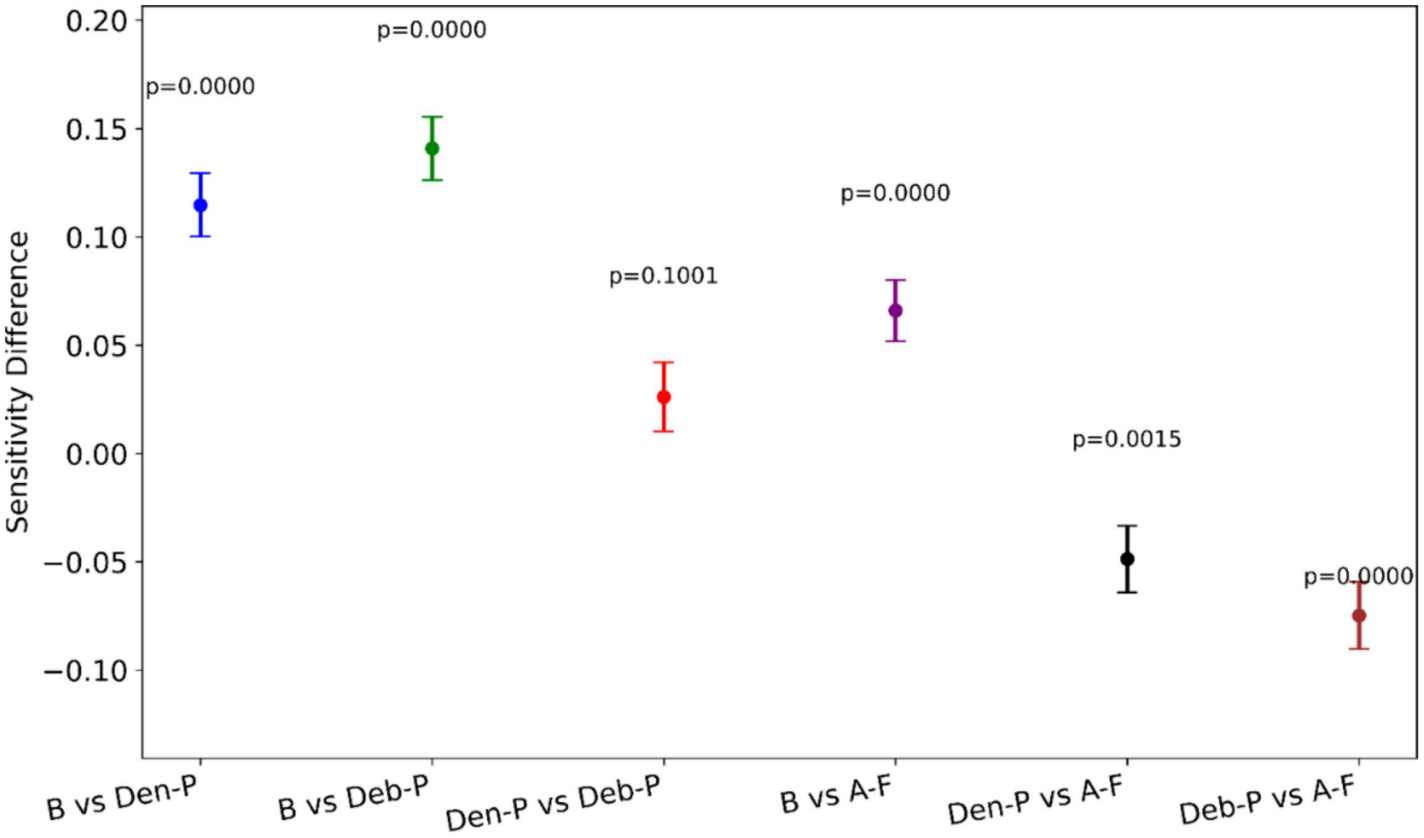
A visual representation of the difference in sensitivity between model pairs alongside the statistical significance of these differences.

Figure 7 provides a visual representation of the difference in the sensitivity between model pairs alongside the statistical significance of these differences. Notable observations from Figure 7 include the following:

- (*B* vs. *Den-P*): There is a significant increase in sensitivity when using the *Den-P* model over the *B*, as evidenced by a positive sensitivity difference and *p* < 0.05.
- (*B* vs. *Deb-P*): Similar to the *Den-P* model, the *Deb-P* model significantly outperforms the *B*, again indicated by a positive sensitivity difference and *p* < 0.05.
- (*Den-P* vs. *Deb-P*): The comparison between the *Den-P* and *Deb-P* models shows a smaller, non-significant difference in sensitivity (*p >* 0.05), suggesting that while both are improvements over the *B*, they may not differ significantly from each other in terms of sensitivity.
- (*B* vs. *A-F*): The *A-F* ensemble exhibits the largest increase in sensitivity compared to the *B*, with *p* < 0.05, indicating a substantial performance improvement.
- (*Den-P* vs. *A-F*) and (*Deb-P* vs. *A-F*): Both comparisons show that the *A-F* ensemble significantly surpasses the *Den-P* and *Deb-P* models (*p* < 0.05).

The *p*-values denote that except for the comparison between the *Den-P* and *Deb-P* models, all other model pairs show statistically significant differences in sensitivity. The consistently low *p*-values reinforce the statistical robustness of the performance gains observed with the models, particularly the proposed *A-F* ensemble.

### 3.4 Activation visualization

**Figure 8:**
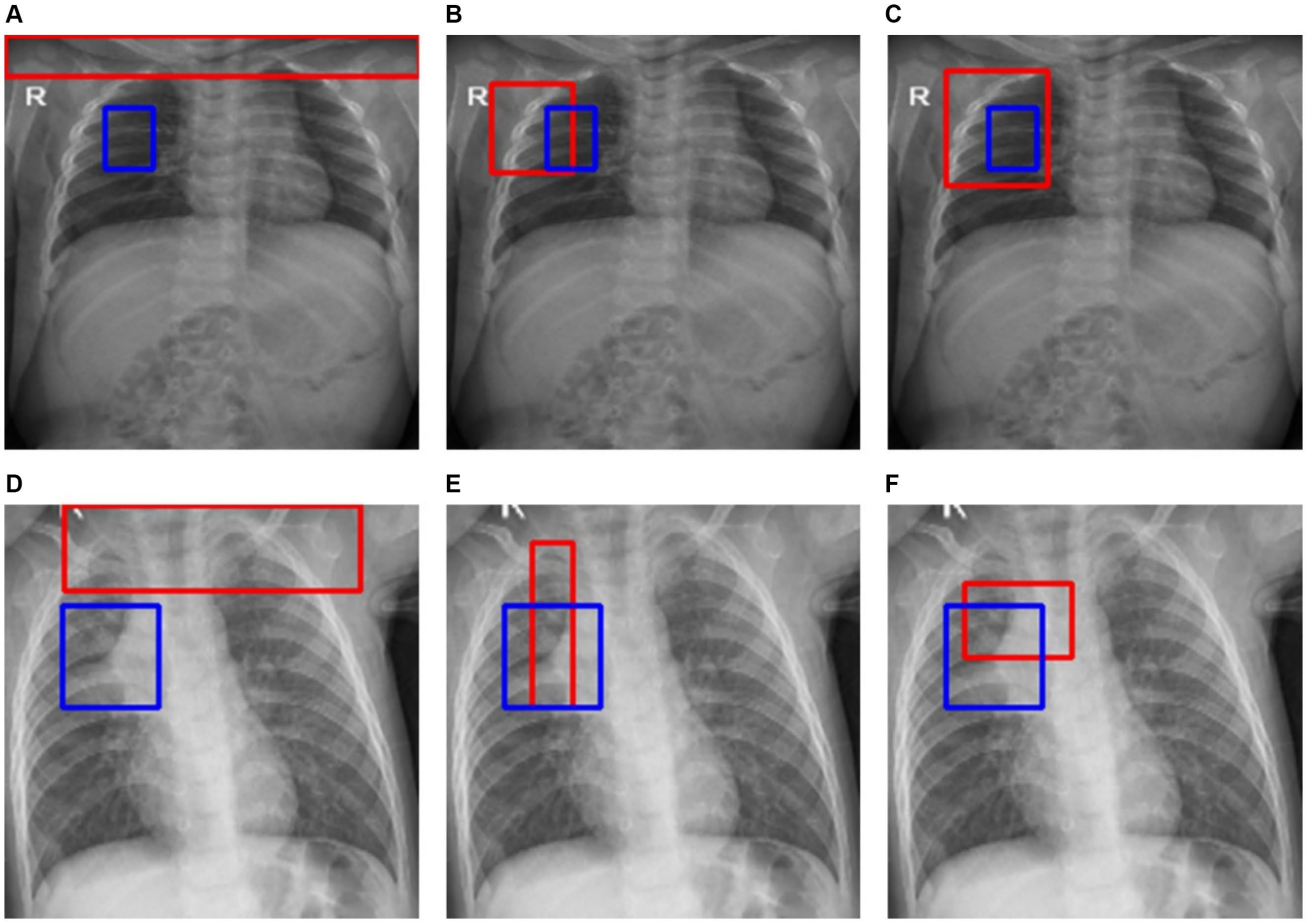
Score-CAM activations (in red bounding box) showing the learned behavior of the models. Blue bounding boxes denote GT annotations. The first row shows a CXR of a pediatric patient with peri-broncho-vascular interstitial opacity in the right middle lobe. The second row shows a CXR of a pediatric patient with atelectasis in the right middle lobe. The sub-figures **(A)** and **(D)** (first column) show the predictions of the *baseline (B)* model. The sub-figures **(B)** and **(E)** (second column) show the predictions of the *Den-P* model. The sub-figures **(C)** and **(F)** (third column) show the predictions of the *Deb-P* model.

Figure 8 shows the Score-CAM activations (in red bounding box) compared with the GT annotations (in blue). The first row shows a text CXR of a pediatric with peri-broncho-vascular interstitial opacity and the second row shows a test pediatric CXR with atelectasis in the right lung. Firstly, we observe the *baseline (B)* model activations in Figures 8A,D cover a broader area and are not tightly focused on the expert GT annotations. Such a lack of precision suggests that the *B* model may not be highly sensitive to the specific clinical features associated with the pathologies mentioned. The *Den-P* model, presented in Figures 8B,E, shows a narrower focus in its activations. Notably, the red boxes are more congruent with the expert annotations, suggesting that the denoising process in the model’s training has potentially led to a refined feature representation. This could be due to the model learning to disregard noise, focusing on the salient features that contribute to the diagnosis. Such precision is likely to increase the sensitivity of the model, as evident from Table 3. The *Deb-P* model’s activations, illustrated in Figures 8C,F, exhibit a different pattern. While the red boxes seem to be concentrated around the areas of expert annotations, they also appear to expand slightly beyond the blue boxes. This suggests that the *Deb-P* model might be picking up on subtler changes in texture or contrast that are not immediately apparent to the human eye but are nonetheless relevant. Such an expansion of activation zones could indicate a heightened sensitivity, potentially leading to higher TP rates, as evident from Table 3. Comparatively, the *Den-P* and *Deb-P* models appear to outperform the *B* model in terms of their activation alignment with expert annotations.

## 4 Conclusion

Our work introduces significant advancements in the application of DL to pediatric CXR analysis. Unlike existing studies that often rely on generic training on large datasets like ImageNet, our study leverages modality-specific pretext learning to address the unique challenges presented by pediatric CXRs. This not only improves accuracy but also enhances the clinical relevance of the models.

Our methodology leverages specialized knowledge from CXR denoising and deblurring tasks to enhance classification performance. By training models to first understand and correct these specific image artifacts, which are prevalent in pediatric CXRs, we effectively enhance the model’s ability to focus on relevant pathological features during the classification task. This approach was demonstrated to be superior to conventional transfer learning (TL) methods, as our results indicated a marked improvement in performance metrics, confirming that our modality-specific pretext learning tasks provide a solid foundation for accurate disease identification.

We adopted the U-Net architecture, traditionally used for biomedical image segmentation, to perform the denoising and deblurring of pediatric CXR images. This adaptation showcases the versatility of the U-Net model beyond its conventional applications, successfully applying it to tasks that require precise artifact correction before classification. The effectiveness of this approach was evident in the improved image quality observed in denoised and deblurred CXRs, which subsequently facilitated more accurate disease classification, underscoring the adaptability and utility of U-Net in new domains of medical image processing.

Our study introduces the novel A-F ensemble, which uniquely combines the outputs of pretext-learned models using a learnable fuzzy-based logic. This innovation was motivated by the need to enhance model reliability and accuracy, particularly in scenarios with ambiguous or conflicting signs in pediatric CXRs. This approach was further validated to demonstrate that the A-F ensemble significantly outperforms traditional ensemble techniques by producing more accurate and stable classification results, as evidenced by improved performance metrics.

Moreover, our novel A-F ensemble approach synthesizes the strengths of individual models through a sophisticated fuzzy logic-based attention mechanism, setting a new benchmark in ensemble learning strategies within medical imaging. This is particularly important as it addresses the variability and often contradictory nature of pediatric CXR interpretations, providing a more robust and reliable diagnostic tool. Additionally, the application of Score-CAM-based activation mapping in our study not only aids in visualizing what the models are focusing on but also confirms that our modality-specific trained models prioritize medically pertinent features more effectively than traditional approaches. This enhanced focus is directly correlated with the observed improvements in classification performance, advocating for a shift toward modality-specific pretraining paradigms in medical imagery.

Despite these promising developments, our research acknowledges certain limitations. Our study concentrates on a binary classification framework and two specific pretext-learning tasks. Expanding the spectrum of pretext tasks could potentially lead to broader and more diverse feature representations, enhancing the model’s diagnostic interpretability. The integration of additional tasks, each uncovering unique aspects within the imaging data, may further elevate the model’s analytical depth. Exploring a wider range of pretext tasks promises to improve model performance not only for CXRs but also across other medical imaging modalities, including MRI and CT scans. Moreover, incorporating contrasting learning paradigms, both supervised and unsupervised, could serve to refine these improvements further.

Regarding the visualization of our models’ behavior via Score-CAM, we must emphasize that while the activation maps yield valuable insights into the decision-making process of the models, translating these observations into clinical relevance necessitates extensive validation. Such validation should involve diverse datasets, encapsulating the full range of targeted pathologies and reflecting demographic variabilities. While our current focus is on binary classification, the true potential of medical modality-specific knowledge transfer lies in its application to more complex scenarios, including multiclass, multi-label, and multi-modal tasks. The extension of our proposed methodology to these areas could substantially push the boundaries of medical diagnostics, leveraging the distinctive diagnostic capabilities inherent in various imaging modalities to gain a more comprehensive understanding of pathologies. To conclude, our findings do more than just highlight the advantages of CXR modality-specific pretext learning; they pave the way for innovative avenues in medical image analysis. They suggest that DL, particularly when tailored to specific medical modalities, holds immense promise for transforming diagnostic practices.

## Data Availability

Publicly available datasets were analyzed in this study. This data can be found at: specified in the manuscript.

## Footnotes

^1^ https://setosa.io/ev/image-kernels/
